# Digitally delivered contingency management during methadone treatment for people with co-occurring cocaine and opioid use: a protocol for a randomized controlled trial

**DOI:** 10.3389/fpsyt.2025.1576277

**Published:** 2025-07-02

**Authors:** Karen Alexander, Anjalee Sharma, Jesse B. Fletcher, Zoe Smith, Tori Huddleston, Jan Gryczynski, Maxine Stitzer

**Affiliations:** Social Research Center, Friends Research Institute, Baltimore, MD, United States

**Keywords:** opioid use disorder, cocaine, contingency management, digital therapeutic, methadone

## Abstract

**Background:**

People in treatment for opioid use disorder (OUD) who also use cocaine are at heightened risk of early treatment discontinuation and poor outcomes. Digital therapeutics utilized within OUD treatment have the potential to impact public health by improving the evidence-based intervention delivery. Contingency management (CM) is an evidence-based intervention to reduce substance use, including cocaine. This article details a randomized controlled trial protocol that tests a digitally-delivered CM intervention for adult patients with co-occurring cocaine and opioid use entering methadone treatment.

**Methods:**

This study will enroll 240 individuals within two weeks of beginning a new treatment episode at one of four participating opioid treatment programs (OTPs) in Baltimore, Maryland. Eligible adults must report past 30-day cocaine and opioid use at intake. Participants are randomized to receive either treatment as usual (TAU) or TAU plus the DynamiCare Contingency Management (DCM) app. The DCM app facilitates the provision of small monetary incentives for picking up methadone doses, completing cognitive behavioral therapy educational modules, and testing negative for cocaine or opioids on randomly-scheduled self-administered and validated oral fluid drug tests. The primary outcome is retention in methadone treatment. Participants are assessed at baseline, 3-, 6- and 12-months on measures including substance use and quality of life, and qualitative interviews are conducted with a subset of DCM-arm participants to assess acceptability and utility of the intervention. This trial was registered in the National Clinical Trials database on February 23, 2023, according to NIH policy (https://clinicaltrials.gov/study/NCT05766631).

**Discussion:**

This project will inform the ancillary content of methadone maintenance treatment for the many patients with substance use disorders that extend beyond opioids, specifically for those who use cocaine. The study’s design will provide scientifically valid information about the effectiveness of digitally delivered CM, while app usage data and qualitative data from participants will be a rich resource for interpreting outcome results. CM is the only known evidence-based treatment for stimulant disorder, and digital delivery could solve many of the practical issues that have hampered widespread adoption of CM. Thus, the study could have both an important scientific and public health impact.

## 1 Introduction

### 1.1 Polysubstance use within opioid use disorder treatment

Methadone is an effective treatment for opioid use disorder (OUD) available in the United States through Opioid Treatment Programs (OTPs). Polysubstance use, or the concurrent use of more than one drug, is common among individuals with OUD. Approximately one-third of people entering treatment for OUD test positive for cocaine use, and cocaine use during methadone treatment is associated with decreased treatment retention and return to opioid use (1). As the pharmacological benefits of methadone treatment are opioid-specific, patients entering treatment for OUD with co-occurring cocaine use may benefit from adjunctive intervention(s) targeting cocaine use (2, 3) to improve their overall treatment retention. Psychological and/or behavioral interventions are applied within OTPs to address cocaine and other substance use disorders during methadone treatment, with varying results (4–6).

### 1.2 Contingency management

Contingency management (CM) is the most effective evidence-based behavioral intervention available to improve stimulant use outcomes, including abstinence from use for up to one year after treatment (7). CM is based on positive reinforcement behavioral techniques and provides a preset reward schedule for successfully attaining specific targeted behaviors (8). In the context of substance use disorder, the targeted behavior is almost universally negative toxicology samples indicating recent drug abstinence, and reinforcements are either chances to receive a prize (‘the fishbowl technique”) or a monetary incentive (9). In order to increase effectiveness and sustain results, reinforcements should be immediate and tied specifically to a verifiable target behavior. There are no other proven treatments for cocaine use disorder (CUD) (10, 11). A large meta-analysis of 157 clinical trials found that only CM programs were associated with a reduction in cocaine use based on objective data from urine tests (10).

However, fewer than 10% of all OTPs implement CM within methadone treatment due to lack of familiarity, lack of resources to provide incentives, and/or the need for ongoing organizational support (12). CM delivery is resource intensive, requiring not just incentives for targeted behaviors, but also considerable staff time to track and administer such incentives properly. Innovative approaches able to reduce such logistical barriers would therefore reduce some of the barriers to OTPs providing CM. Digital tools like smartphone applications can increase the reach of evidence-based interventions while simultaneously handling many of the mundane-but-necessary tasks associated with CM (e.g., tracking reward schedules and administering incentives), thereby potentially bridging the current implementation gap. Mobile health (mHealth) technologies are increasingly studied as innovative ways to bring CM interventions to substance use treatment settings (13), with the expectation that such technology-based interventions and monitoring tools could improve patient engagement and retention, reduce provider burdens, and optimize outcomes (14–16).

Existing data from both patients and OTP providers reveal high enthusiasm for and engagement with mHealth-based adjunctive treatments that include self-administered cognitive-behavioral lessons, treatment reminders, incentives, and assessments (17–19). Patients receiving mobile intervention content during methadone treatment report higher satisfaction with their treatment experience than patients who do not (19), and providers report appreciating the time saved via mobile interventions’ automation of several aspects of patient monitoring (17). Preliminary evidence also suggests that people in methadone treatment who are provided access to mobile intervention content may outperform patients receiving only treatment-as-usual in both opioid abstinence and treatment retention (19). However, clinical research using digital platforms to deliver CM predominately focuses on cigarette smoking and alcohol use (20–22). This study is the first to use a digital platform to deliver CM with the aim of impacting methadone treatment retention by reinforcing abstinence from polysubstance use, specifically cocaine use.

### 1.3 DynamiCare health

DynamiCare Health Contingency Management (DCM) is a commercially available mHealth application that automates several aspects of evidence-based CM by assigning and remotely verifying achievement of targeted behaviors, tracking reward schedules, and ensuring near-immediate delivery of rewards. Drug testing via photo- and video-captured oral fluid swabs can occur in any location that works best for the participant (e.g., work, home, school), increasing convenience and acceptability across various life circumstances. Evidence suggests the DynamiCare app is highly acceptable to patients and providers, simplifying the acts of earning and spending rewards for patients and easing the burdens of CM administration for providers (23). Satisfaction with the app among participants is consistently high, and participants report enjoying being engaged and in better control of their own recovery process (24). In clinical trials, the DynamiCare app has been shown to significantly improve appointment attendance among opioid- and alcohol-using populations and has been associated with significant increases in the odds of both substance abstinence and medication adherence (23, 25). The DCM app also includes cognitive behavioral therapy modules reinforcing recovery support and coping skills.

## 2 Methods

### 2.1 Study design and overview

This protocol is a two-arm, parallel randomized controlled trial comparing methadone treatment as usual (TAU) versus TAU plus exposure to the DCM app (TAU+DCM). The target sample consists of 240 adult patients with co-occurring cocaine use entering methadone treatment in the community. The traditional DCM program is customized to target two primary behaviors with CM: 1) abstinence from opioids and cocaine as verified via remote oral fluid testing, and 2) medication pickup from the methadone program as verified by clinic records. Randomization must occur within two weeks of a new treatment episode beginning at the participating OTP. Participants randomized to TAU will receive standard methadone treatment at their programs. Participants randomized to TAU+DCM will receive standard care at the methadone program and access to the DynamiCare Health app. For 48 weeks post-randomization, participants in the TAU+DCM arm will have continual access to the app while they are in methadone treatment, including access to CBT learning material and the opportunity to earn cash incentives for drug-negative tests and for medication pick-up. The primary outcome of retention in methadone treatment will be tracked through clinic records. Participants will be assessed at baseline (randomization), 3-, 6-, and 12-months post-randomization on opioid, cocaine, and other untargeted substance use; quality of life; and perceived utility of the app as a recovery tool for polysubstance use (among those randomized into the DCM arm only). Aims of the trial are listed below.

*Aim 1* of the study is to determine the relative effectiveness of TAU+DCM compared to TAU alone in improving methadone treatment retention through 12-months post-treatment entry.

*Aim 2a* of the study is to determine the relative effectiveness of TAU+DCM compared to TAU alone in reducing opioid use and cocaine use through 12-months post-treatment entry.

*Aim 2b* of the study is to determine the relative effectiveness of TAU+DCM compared to TAU alone in terms of improving other secondary outcomes, including non-targeted substance use and quality of life through 12 months post-treatment entry.

*Aim 3* will explore DMC app use patterns, acceptability, and perceived value of DCM app content through 12 months post-treatment entry.

Our overarching hypothesis is that participants in the TAU+DCM arm will have longer treatment retention, lower rates of opioid and cocaine use, and superior secondary health outcomes compared to participants in the TAU arm.

### 2.2 Trial registration and data and safety monitoring

This trial was registered in the National Clinical Trials database on February 23, 2023, according to NIH policy (https://clinicaltrials.gov/study/NCT05766631). WCG IRB approved the study and provides oversight (Protocol ID: #36419122.1). A federal Certificate of Confidentiality was automatically issued as a part of the NIH grant award. An independent Data Safety Monitoring Board monitors the study and reviews all adverse events annually, or more often if needed.

### 2.3 Data collection

#### 2.3.1 Study sites

The original intention was to recruit from two OTPs in Baltimore City, but the study expanded to a total of four OTPs quickly because of initial challenges in recruitment. The four participating OTPs are CARF-accredited and have a long history of research partnership with the study investigators.

#### 2.3.2 Recruitment and eligibility

New patients are recruited through IRB-approved study flyers and clinician referral to an on-site research assistant. Research Assistants (RAs) are also notified by the clinic when a new client enters treatment and clinic staff introduce the patient to the RA, who invites them to be screened for eligibility if interested. RAs meet with the patient privately, either in-person or by phone, to discuss the study and to complete eligibility screening. The study has the following inclusion criteria (1): adult (ages 18 and older) clients entering methadone treatment at one of the participating programs; (2) cocaine use (self-reported in the past 30 days); and (3) willing and able to provide informed consent. Study exclusion criteria are: (1) severe psychiatric disorders (e.g. active suicidality or hallucinations); (2) on home detention; (3) visual impairment that would make the use of the phone app impractical; and (4) living arrangement that currently restricts phone access.

#### 2.3.3 Informed consent

During the baseline interview, prior to randomization, the RA reviews the IRB-approved informed consent documents that describe both study arms and explains the risks and benefits of participation. To assess understanding, RAs administer a brief consent quiz on which individuals must receive a perfect score within three attempts to be deemed eligible to participate in the study.

#### 2.3.4 Randomization

Following informed consent and baseline assessment, during which research staff and participants are blind to condition, participants will be randomized to TAU or TAU+DCM in blocks based on three prognostic factors: (1) age (< 50 vs. ≥ 50; binary); (2) recent cocaine use (daily vs. non-daily use at intake; binary); and, (3) gender (man vs. woman; binary). The adaptive allocation approach will ensure that the study arms are balanced with respect to these three prognostic variables. Age is included due to concerns that participants older than the age of 50 may be less familiar with smartphone apps than younger participants; cocaine use frequency is included to ensure that people who use cocaine daily were not over-represented in one condition over the other; and, gender is included due to known gender differences in expected response to methadone treatment (26–28).

#### 2.3.5 Study conditions

Participants randomized to the TAU Condition receive standard treatment at the methadone clinic, which includes daily medication and, depending on the clinic and level of care/need, individual and/or group addiction counseling. Participants in the TAU condition represent the real-world status quo that patients would experience if they did not participate in the study. After admission into the program, new patients complete the program’s psychosocial and medical intake, methadone dose induction and adjustment, provide urine tests, and receive medication take-homes, if eligible, according to each programs’ policies and methadone treatment regulations.

Once a participant is randomized to TAU+DCM, the RA downloads the DCM app onto the participant’s personal smart phone (or a study-provided phone if the participant does not own a smartphone). Participants can receive up to two study-provided phones and data if needed. Participants in the DCM arm complete an orientation session with the RA, beginning with an introduction to basic smart phone functionality, including how to open and close apps, lock and unlock the phone, charge the phone, and other fundamental smartphone functions. The RA then walks the participant through opening and checking the functionality of the DynamiCare Health app, giving the participants the oral fluid testing kits, and registering the participant with a PEX debit card upon which any earned CM rewards will be loaded (see below). Funds can be added to PEX cards remotely, and the cards cannot be used at certain types of businesses (e.g., liquor stores). Participants are trained to use the oral fluid test kits by the RAs, practicing until they can produce two valid tests in a row, one without aid of the research staff. TAU+DCM participants earn $10 for successful completion of the orientation and training. Participants are given a hard copy of a brief manual regarding the phone (if applicable) and DCM app operations. Orientation is typically accomplished within 3–5 days of randomization. One week into the study the RA checks in with the participants and provides additional training on the functionality of the app, if needed. A brief refresher training is offered to all TAU+DCM participants again at 4 weeks.

Drug testing during the intervention is accomplished by participants using 16-panel oral fluid (saliva) test kits (Premier Biotech) (fentanyl, tramadol, ethanol alcohol, synthetic cannabinoids, morphine, oxycodone, marijuana, cocaine, benzos, amphetamines, methamphetamines, buprenorphine, methadone, phencyclidine, ecstasy, barbiturates). However, as the intervention’s target behaviors are abstinence from cocaine and illicit opioid use, participant incentives are based only on the “cocaine” and non-treatment “opioid” test strip results. Prompts to begin a self-administered test are sent under a random schedule starting at approximately twice weekly and gradually reducing in frequency to once weekly for participants who achieve consistent strings of tests showing abstinence from opioids and cocaine. Participants can be pinged for oral fluid testing anytime within a 12-hour window of their choosing. Once pinged, participants have 4 hours to fulfill oral fluid testing. Timeframes can be adjusted to accommodate participant schedules, but participants must pick timeframes that total 12 hours in a 24-hour period. Oral fluid testing can be pinged at the end of the 12-hour window, and participants would have 4 hours to complete the oral fluid testing, which could then fall outside of the 12-hour window. If a test is missed, the participant is alerted that a test has been missed and that another one will be sent at a random time in the future. Participants are not penalized for missing oral fluid tests.

Oral fluid drug test validity is verified by a trained DynamiCare staff member who receives video and/or photo display(s) of both the test process and result directly from the participant. Both pictures and videos are taken during the oral fluid testing process, which can take 2–5 minutes, on average. Both are usually needed to confirm validity of the testing; the video shows the entire process of oral swab testing (e.g., confirming correct participant, method of testing, etc.), while the picture is used to validate the findings from the swab (i.e., a close-up picture of the swab with results).Once test results are verified, the DCM app automatically calculates the appropriate reward and delivers that reward directly to the participant’s personal PEX debit card; specific rewards amounts are determined using an escalating schedule which increases incentive values provided per “consistent” test while also decreasing the likelihood of receiving another test that same week; concurrent escalation of reward value and deceleration of testing frequency balance incentive earnings at ~$10.50 per week. Methadone pick-up is confirmed by RA review of clinic records, with weekly disbursement of rewards onto the PEX card at $10 per week if all scheduled methadone pickups are successfully completed; two dollars is subtracted for each missed dose during a single week. If more than a single week of medication has been disbursed (i.e., “take home” doses), the reward payment continues until next scheduled pickup is verified. Finally, the app provides 72 self-administered CBT modules that are designed to aid in relapse prevention; small incentive rewards (i.e. $1) are provided for completion of up to 3 modules per week for the first 24 weeks post-enrollment. A maximum of $1066 can be earned over 48 weeks if a TAU+DCM participant accomplishes all targeted behaviors. The incentive structure is illustrated in **Table 1**.

**Table 1:** Incentive structure for target behaviors using DynamiCare app.

Target	Study Week (*4-week Payment Intervals*)												Total
	1 thru 4	5 thru 8	9 thru 12	13 thru 16	17 thru 20	21 thru 24	25 thru 28	29 thru 32	33 thru 36	37 thru 40	41 thru 44	45 thru 48	
Opioid & Cocaine Abstinence	$42	$42	$42	$42	$42	$42	$42	$42	$42	$42	$42	$42	$504
Methadone Pick-Up	$50	$40	$40	$40	$40	$40	$40	$40	$40	$40	$40	$40	$490
CBT Module Completion	$12	$12	$12	$12	$12	$12							$72
Total	$104	$94	$94	$94	$94	$94	$82	$82	$82	$82	$82	$82	$1,066

### 2.4 Operational definitions

In year two of recruitment, the study team became aware of the need to clarify and refine the protocol’s operational definitions. As the purpose of the study is to measure retention in methadone treatment and compare outcomes between study groups, it is necessary to define a “new patient” accurately. This was more challenging in practice than originally anticipated in preparation for the trial. In the end, we operationalized a “new patient” as someone starting a new treatment episode. Therefore, transfers from another program were excluded. Determining who was entering a new treatment episode necessitated both historical data elicited from the participant, review of the OTP’s electronic health record, and an investigation into methadone dosing upon entry to a study site. Patients were deemed ineligible if intake documentation indicated transfer from another program. Participants with starting doses higher than 50 mg were subjected to additional review to ensure they were not receiving methadone elsewhere in the recent past.

### 2.5 Assessments

After completing the informed consent but prior to randomization, participants provide detailed locator information and complete the baseline research assessment. The in-person assessment includes self-report measures (sociodemographic characteristics, living situation(s), DSM-5 substance use disorder criteria Addiction Severity Index (ASI), and the World Health Organization Quality of Life Measure (WHOQOL-BREF). The ASI will gather detailed information on frequency, route, and timing of alcohol and drug use over the past 30 days and 6 months. Participants will report daily use, substances used, timing, and order, followed by questions to identify polysubstance use patterns (e.g., substitution, replacement, complementing/countervailing, contamination). Polysubstance use will be assessed at baseline and follow-ups via self-report questions covering typical and atypical use, time of use, and reasons for combining substances. Quality of life will be measured using the WHOQOL-BREF, which provides scores (0–100) across four domains: physical, psychological, social, and environmental. Follow-up assessments are conducted at 3-, 6-, and 12-months post-randomization; participants in both arms receive $50 for each assessment they complete, including baseline (maximum earnings for research assessments = $200). Urine testing for substance use only occurs at follow-up. Urine samples will be collected by project staff at research at each follow-up visits, either at the OTP or at the research office, and tested using 16-panel point-of-care assays for morphine metabolite, oxycodone, fentanyl, tramadol, methadone, buprenorphine, cocaine, benzodiazepines, amphetamine, methamphetamine, barbiturates, phencyclidine, MDMA, THC, synthetic cannabis, and ethyl glucuronide (alcohol). Results will be used for research purposes only and will not be shared with clinic staff. In addition, some intervention participants are selected based on app use and randomization characteristics to complete semi-structured, qualitative interviews at follow-up time points. Participants receive an additional $50 for completing each qualitative interview. A summary of measures and the initial planned analysis strategy is shown in **Table 2**.

**Table 2:** Data sources and assessment schedules.

Outcome Variable	Measure	Data Source	Assessment Time Points	Endpoint or Repeated	Distributional Assumption	Statistical Model
Primary Outcome						
# Continuous Days in Methadone Treatment	Count	Clinic Records	Daily thru 12m	Endpoint	Survival	Cox PH regression
Secondary Outcomes						
Urine Screening: Opioids	Urine Test	Point-of-care Immunoassay	Baseline, 3-/6-/12-month Follow-up	Repeated	Binomial	GLiMM
Urine Screening: Cocaine					Binomial	
Urine Screening: Other Drug(s)					Binomial	
Frequency of Opioid Use (past 30 days)	ASI	Self-report	Baseline, 3-/6-/12-month Follow-up		Poisson or Negative Binomial	
Frequency of Cocaine Use (past 30 days)					Poisson or Negative Binomial	
Frequency of Other Drug Use (past 30 days)					Poisson or Negative Binomial	
Familial, Legal, Medical Issues					Continuous Non-Parametric	
Quality of Life	WHOQOLBREF				Continuous Parametric	
Exploratory Outcomes						
App Usage Patterns	Backend and Metadata	DynamiCare	Daily thru 48w	Endpoint	Continuous Parametric	GLiMM
Incentive Rewards Earned			Daily thru 48w	Endpoint	Continuous Parametric	
Oral Swab Testing			Daily thru 48w	Repeated	Binomial	
Engagement with CBT Modules			Daily thru 48w	Endpoint	Poisson or Negative Binomial	
Incentive Spending	PEX Debit Card		Daily thru 48w	Endpoint	Continuous Parametric	

## 3 Outcomes

The primary outcome is retention in methadone treatment which will be determined through clinic records. Drop-out is defined as seven consecutive days without picking up a scheduled methadone dose (after any previously provided take home doses have run out, if applicable). Participants who are temporarily hospitalized or incarcerated are considered “retained to treatment” if they continue to be dosed and return to treatment within seven days after release. However, in these settings, participants are not incentivized for methadone pick-ups as they cannot demonstrate the target behavior of picking up their methadone dose. This is a reasonable strategy, as both acute care facilities and state carceral institutions offer continuation of methadone for individuals who are in treatment at the time of hospitalization or arrest, if the patient notifies them of their methadone treatment status. Such situations are routinely documented within the clinic records, as the OTP must verify doses with the hospital or carceral institution. Secondary outcomes of this study include reductions in opioid, cocaine, and other drug use; improvements in participants’ quality of life, familial, legal, and medical issues.

## 4 Qualitative interview data

Qualitative data is collected throughout the clinical trial to aid in the understanding of the role of the DCM app in the treatment and early recovery process from the patient’s perspective. Semi-structured qualitative interviews are digitally recorded, transcribed verbatim (omitting participant names and other potentially identifying information), checked for accuracy, and uploaded to NVivo to aid with analysis. Qualitative data analysis for this study will be analyzed for three separate purposes: 1) to explore anticipated characteristics of app use patterns, acceptability, and perceived value; 2) to explore unanticipated characteristics of app use patterns, acceptability, and perceived value; and 3) to inform interpretation of quantitative data once analyses are complete.

## 5 Statistical analyses

All analyses will be conducted on available study-related data from all participants, regardless of whether or when they drop out of treatment. Likewise, the primary analysis will be conducted based on the conditions to which participants were assigned, regardless of the level of adherence to the intervention. The primary outcome of retention in methadone treatment will be examined as a time-to-event phenomenon using Cox proportional hazards regression. A Generalized Linear Mixed Model (GLiMM) will be used to conduct analyses of secondary outcomes. Additional supplementary analyses will examine outcomes on the basis of app engagement or DCM intervention ‘dose’. Backend and metadata from the DynamiCare app will be reviewed continuously through the intervention, including information regarding app usage patterns (including participants’ engagement with the optional CBT modules), incentive rewards as a measure of “intervention dose”, oral swab testing, and incentive spending habits (e.g., as an indicator of participants’ engagement with the reward system). See **Table 2** for details on data sources and assessment schedules for all primary, secondary, and exploratory outcomes.

Outcome variables will be of four distinct types: 1) time-to-event data (*primary outcome:* treatment retention through 12 months); 2) dichotomous variables (*secondary outcomes:* illicit opioid and cocaine urine test results over time, meeting OUD and CUD criteria), assumed to follow a binomial distribution; 3) discrete random variables (*secondary outcomes:* number of days of opioid and cocaine use in the past 30 days), assumed to follow a Poisson distribution [will be tested against negative binomial distribution using the Wald test]; and, 4) continuous random variables (*secondary outcomes:* WHO QoL- BREF scores; ASI composite scores), assumed to follow a normal distribution. All distributional assumptions will be evaluated prior to conducting all analyses, and if such assumptions are not met, assumptions will be modified and statistical methods chosen accordingly, and/or outcome measures transformed appropriately.

**Power Analysis**. Power for the study’s primary outcome (i.e., time to treatment discontinuation) was determined using Cox proportional hazards regression using SAS PROC POWER. Assuming N=240, α=0.05, the standard deviation of the Study Arm=.5, the multiple correlation between the covariates and the Study Arm equal to 0 (given covariate adaptive randomization), and using an overall treatment discontinuation rate of 50% within one year (with the remaining participants staying in treatment and considered censored), power to detect a difference between the TAU and TAU+DCM arms exceeds.82, corresponding to a hazard ratio of 1.7 (a small-to-moderate effect size). Even smaller effects can be detected with power >.80 if the overall treatment discontinuation rate is higher. If the overall rate of treatment discontinuation is lower (40% leaving treatment within one year), power to detect a hazard ratio of 1.8 would exceed.82. Thus, with the planned 240 participants, the study will be well-powered for detecting relatively small but clinically meaningful differences in the primary outcome of retention.

## 6 Discussion

This study is the first randomized controlled trial to apply digitally-delivered CM to improve treatment retention among methadone clients being treated for OUD who concurrently use cocaine (20). Evidence generated from this study may inform the future delivery of methadone treatment, as it could demonstrate the ability of a digitally-delivered CM intervention to support treatment retention among patients with co-occurring cocaine use, a factor commonly associated with early dropout from methadone treatment. Digital-delivery of CM brings innovation to substance use treatment through its versatile platform and consistent structure, allowing both drug testing and incentive delivery in the community. Digital-delivery has flexibility, permitting incentives for a range of recovery-oriented behaviors relevant to treatment retention, including engagement with CBT modules and abstinence from target substances (29).

This protocol describes an innovative and effective strategy that could be integrated within the existing national OTP service delivery system to more effectively address polysubstance use involving opioids and stimulants, making it simultaneously novel and practical. The current lack of OTPs using CM may stem from logistical complexity and resource limitations for evaluating targeted behaviors and delivering incentives, which digital delivery could address. In the current study, initial recruitment challenges led to expanding the study recruitment sites from two to four OTPs – demonstrating openness to the intervention across several study sites.

The financial viability of new therapies, even if they are shown effective, is always an issue. Insurers, including Medicare and Medicaid, are expanding coverage of digital therapeutics so that CM programs like DynamiCare are well-positioned to enhance treatment programs in a scalable, sustainable way (30). Additionally, growing national support for CM has sparked increased interest in applying this evidence-based approach in OTPs (29). More insurers and health systems are covering CM treatments, recognizing the value of incentivizing healthier behaviors, with some states even reimbursing for CM-based interventions (31, 32).

However, the recruited population experiences ongoing challenges, including economic deprivation, as well as high levels of housing instability amid a complex and dynamic service environment with respect to the intersection of OUD treatment and housing. These factors may interfere with their ability to effectively utilize digital therapeutics. It is also important to consider patient perspectives about the acceptability and utility of new interventions. Patients are the experts in their own experience and may have useful insights into the methods that can help them with behavior change. Our qualitative data will shed light on the usefulness of the DCM app, and the level at which CM rewards motivate behavior change from the participants’ perspective. Study recruitment is ongoing, with the accrual of the target sample of 240 participants expected in the Spring of 2026.

### 6.1 Limitations

We acknowledge that one limitation of this study is that some participants in the intervention arm may not actively engage with the DCM app for the full 48-weeks, and not receive a full ‘dose’ of the intervention. To address this possible limitation, DCM app usage will be tracked, and we will continue to follow and collect data for the full intervention period regardless of participants’ engagement level or duration. We also recognize the barriers which may exist for our participants, most of whom are experiencing poverty and housing instability, making this type of CM implementation challenging. Participants are asked to perform oral fluid tests in their own environments using testing kits provided to them, at random times throughout the day. However, many participants face challenges in securing their belongings in shelter or group housing settings, which often lack the privacy and autonomy needed to make random oral fluid testing feasible. Our research staff works with participants to replace lost or stolen testing kits and assist participants who lose phones or lack sufficient data to upload video tests to the app. However, we acknowledge that these support measures may not be sustainable beyond the clinical trial setting.

### 6.2 Conclusion

This project could demonstrate the effectiveness of an intervention to improve outcomes in methadone maintenance treatment for the many patients with co-occurring cocaine use, which puts them at elevated risk of treatment dropout. The design of the study will provide scientifically valid information about the effectiveness of digitally-delivered CM, while the observational data on app usage and the qualitative data from participants will be a rich resource for the interpretation of outcome results. This is an important study because CM is the only known evidence-based treatment for stimulant use disorder, and digital delivery could solve many of the practical issues which have traditionally hampered the adoption of CM. Thus, the study could have both important scientific and public health impacts.
